# A Retrospective Comparison of Trastuzumab Plus Cisplatin and Trastuzumab Plus Capecitabine in Elderly HER2-Positive Advanced Gastric Cancer Patients

**DOI:** 10.1097/MD.0000000000001428

**Published:** 2015-08-28

**Authors:** Bo Zhu, Jun-Rong Wu, Xiao-Ping Zhou

**Affiliations:** From the Department of Clinical Laboratory, The Afffiliated Tumor Hospital of Guangxi Medical University, Nanning, Guangxi, China (BZ, J-RW); and Department of Clinical Laboratory, First Affiliated Hospital of Guangxi University of Chinese Medicine (X-PZ).

## Abstract

A combination of trastuzumab and cisplatin or trastuzumab and capecitabine has been confirmed to be effective for treating adverse effects in with HER2-positive advanced gastric cancer (AGC) patients. We retrospectively compared the activity and safety of trastuzumab plus cisplatin (HP) and trastuzumab plus capecitabine (HX) for elderly HER2-positive AGC patients.

Ninety two HER2-positive AGC patients were included in this study; of those 48 patients received trastuzumab (course 1, 8 mg/kg followed by course 2, onward, 6 mg/kg on day 1) plus cisplatin (60 mg/m^2^) intravenously on day 1 of a 3-week cycle and 44 patients received trastuzumab (course 1, 8 mg/kg; course 2 onward, 6 mg/kg) plus intravenous oral capecitabine (1000 mg/m^2^ twice daily on days 1–14), every 3 weeks. The primary end point was overall survival (OS). The secondary end points included objective response rate (ORR), progression-free survival (PFS), and toxicity.

The median age was 71 years in both groups. The median OS was 15.5 months in the HP group and 17.0 months in the HX group, with no significant difference between the 2 groups (*P* = 0.78). There were also no significant differences in PFS (median 6.6 months vs 7.2 months, respectively; *P* = 0.90) and ORR (58.3% vs 59.1%, respectively; *P* = 1.00) between the HP group and the HX group. The major grade 3 or 4 adverse events in the HP group and the HX group were neutropenia (35.4% vs 29.5%, respectively), followed by anorexia (25.0% vs 22.7%, respectively), and anemia (16.7% vs 13.6%, respectively), no significant differences were observed.

HP and HX were associated with similar efficacy and safety in HER2-positive AGC patients.

## INTRODUCTION

Patients with gastric cancer are often diagnosed in an advanced stage, and there has not been a globally accepted standard therapy for advanced gastric cancer (AGC). Thus, AGC remains a common malignancy worldwide that seriously reduces the quality of life of patients, and the prognosis of AGC patients remains poor. In general, the combination of trastuzumab and cisplatin is the chemotherapy regimen that is most frequently used to treat AGC,^1,2^ although patients receiving that treatment experience considerable adverse effects and they endure many inconveniences.^3^ S-1, as a new novel oral fluoropyrimidine agent, has already been confirmed to show promising anticancer efficacy for AGC.^4,5^ Because the S-1 plus cisplatin (SP) chemotherapy regimen is accepted as the standard therapy for AGC in Japan,^6^ this combination has been studied in several completed clinical trials.^7,8^ However, this regimen consistently produces high incidences of grade 3 to 4 hematological toxicities.^9,10^ Currently, more and more clinical research studies have focused on molecular targeted chemotherapy. It is still unknown as to whether human epidermal growth factor receptor 2 (HER2) status influences the survival of patients who receive SP as a first-line chemotherapy.^11^ Therefore, dose optimization and selection of a combination partner are emerging questions in research on chemotherapy agents for AGC patients.

The HER2 protein (p185, HER2/neu, and ErbB-2) is a 185-kDa transmembrane tyrosine kinase (TK) receptor and a member of the epidermal growth factor receptor (EGFR) family. It is strongly associated with increased disease recurrence and a poor prognosis of breast cancer.^12^ Overexpression or amplification of HER2 has also been observed in ovarian and stomach cancer as well as aggressive forms of uterine cancer. Trastuzumab (Herceptin, Roche, Basel, Switzerland) is a recombinant humanized monoclonal antibody that targets HER2, and a one-year period of administration of adjuvant trastuzumab has already been confirmed to be the standard of care in the treatment of patients with early-stage HER2-positive breast cancer.^13^ Trastuzumab-based chemotherapy has been given to HER2-positive AGC patients with promising clinical benefits and acceptable levels of toxicities. A phase II trial indicated that trastuzumab in combination with S-1 plus cisplatin resulted in promising antitumor activity in HER2-positive AGC patients, including a 68% response rate, a 94% disease control rate, and 16.0-month median overall survival (OS). The major grade 3 or grade 4 adverse events included: neutropenia (36%), anorexia (23%), and anemia (15%).^14^ Results from a multicenter phase II study showed that a combination of trastuzumab and XELOX (capecitabine plus oxaliplatin) is highly effective in HER2-positive AGC patients in a South Korea population, including a 67% objective response rate, 9.8-month progression-free survival (PFS), and 21.0-month median OS, as well as tolerable grade 3 to 4 toxicities, although with 1 treatment-related death case.^15^ Treatment using a combination of trastuzumab and cisplatin or trastuzumab and capecitabine was also examined in several completed clinical trials and the results showed promising antitumor outcomes. Although capecitabine and cisplatin are considered to be a first-line regimen for AGC, the differences between trastuzumab plus capecitabine (HX) and trastuzumab plus cisplatin (HP) for treating HER2-positive AGC have not been studied. In addition, most of the relevant research studies were conducted in Japan; therefore, this retrospective study was performed to evaluate the efficacy and safety of first-line chemotherapy with HP or HX in HER2-positive AGC patients in China.

## PATIENTS AND METHODS

### Patients

This is a retrospective study. Ninety two patients who had received HP or HX at our institution were included. The major eligibility criteria for this study were: aged ≥65 years; histologically confirmed AGC, metastatic or recurrent gastric adenocarcinoma; Eastern Cooperative Oncology Group (ECOG) performance status (PS) ≤2; a positive HER2 status, as evaluated using immunohistochemistry (IHC) and fluorescence in situ hybridization (FISH); no previous chemotherapy or radiation therapy except adjuvant chemotherapy completed at least 6 months before study; and adequate bone marrow (3000/μL ≤WBC count ≤12,000/μL; leukocyte count ≥4000/μL; absolute neutrophil count ≥1500/μL and platelets ≥100,000/μL), hepatic (serum transaminase <3 times the upper normal limit; serum bilirubin <1.5 mg/dL), and renal function (serum creatinine <1.5 mg/dL). Patients were excluded if they had a concurrent or prior active malignancy, a serious comorbid condition, brain metastases, or an active infection. This trial was approved by the institutional ethics committee, and all patients gave written informed consent for chemotherapy.

### Treatment Schedule

In the HP group, trastuzumab was administered at an initial loading dose of 8 mg/kg as a 90-minute infusion, followed by 6 mg/kg at 3-week intervals as a 30- to 90-minute infusion. Cisplatin was given by intravenous infusion at 60 mg/m^2^ on day 1, repeated every 3 weeks. Patients in the HX group received trastuzumab (6 mg/kg every 3 weeks following a loading dose of 8 mg/kg on cycle 1) plus capecitabine (1000 mg/m^2^/day, day 1 to day 14, every 3 weeks). Chemotherapy dose adjustments were allowed. Treatment was repeated until the occurrence of disease progression, unacceptable toxicities, the patient's refusal to continue, or death.

### Evaluation of Treatment and Statistical Analysis

The toxicity assessments and laboratory data were assessed weekly. National Cancer Institute Common Toxicity Criteria for Adverse Events version 4.0 were used to assess toxicity. Tumor assessments were carried out after 2 cycles of antitumor agents were administered according to the Response Evaluation Criteria in Solid Tumors guidelines (version 1.1). Patients received computed tomography scans every 3 months. The primary end point was OS (defined as the time from the date of initiation of chemotherapy to the date of either death or the last follow-up visit). The secondary end-points included objective response rate (ORR) (the complete response [CR] rate plus partial response [PR] rate with measurable lesions according to Response Evaluation Criteria in Solid Tumors), PFS (measured from the date of initiation of chemotherapy to the date of progressive disease or death from any cause), and toxicity.

Distributions of the discrete variables were compared between the 2 treatment groups with either the Chi-square test or Fisher exact tests. Nonparametric testing with the Mann-Whitney *U* test was used to compare the continuous variables. Kaplan–Meier analysis with log-rank testing was used for univariate analysis. Variables showing a trend for association with survival (*P* < 0.05) and variables that were known to have prognostic value were selected in the final multivariable Cox proportional hazards model. SPSS software (version 16; SPSS Inc., Chicago, IL) was used for statistical analysis; all tests were 2-tailed and *P* < 0.05 was considered to be statistically significant.

## RESULTS

### Patient Characteristics

**FIGURE 1:**
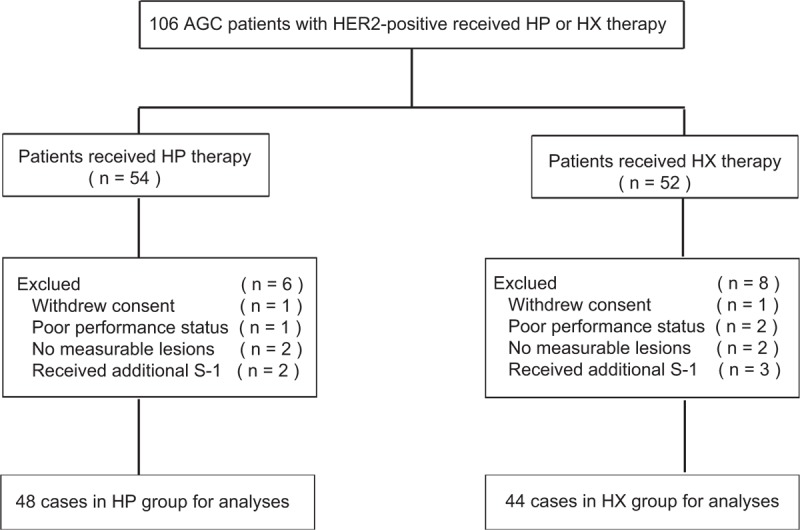
Patient selection: HP group and HX group. HP = trastuzumab plus cisplatin, HX = trastuzumab plus capecitabine.

During the time period of the study, 106 HER2-positive AGC patients from Guangxi Medical University Cancer Hospital were screened for inclusion. Of those 106 patients, 14 were excluded for the following reasons: poor PS (3 patients), withdrawn informed consent (2 patients), insufficient measurable lesions (4 patients), and administration of additional S-1 agent (5 patients). Thus, the remaining 92 patients were divided into 2 groups as follows: 48 patients were assigned to the HP group and 44 patients were assigned to the HX group. Figure 1 shows the flow chart of the patient selection process used in this study. Table 1 shows the characteristics of the eligible patients. The baseline characteristics were well balance between the 2 treatment groups.

**TABLE 1:**
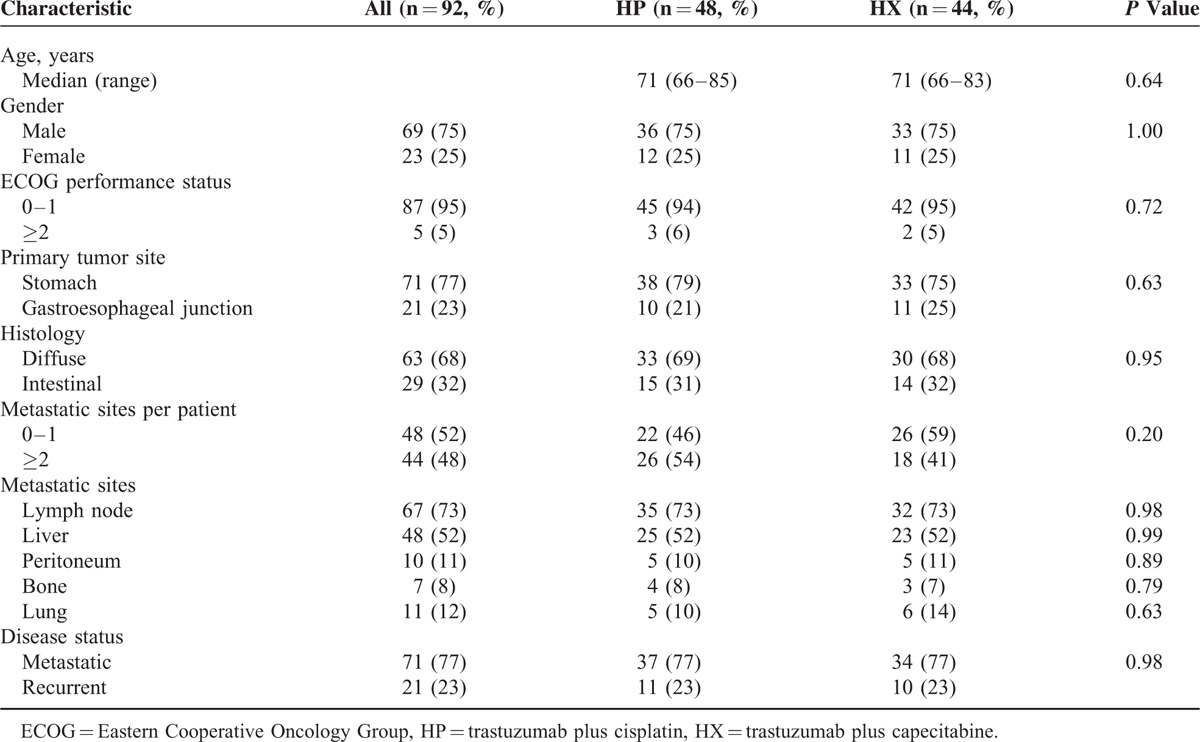
Baseline Characteristics

## TREATMENT RESULTS

### Treatment Cycles and Doses of the Drugs

The median number of treatment cycles was 5 in the HP group and 6 in the HX group. The median duration of follow-up was 15.3 months in both groups. In the HP group, after the first cycle, the dose of trastuzumab was decreased in 16 patients and the dose of cisplatin was decreased in 14 patients. In the HX group, the dose of trastuzumab was decreased in 14 patients, and the dose of capecitabine was decreased in 15 patients. Hematological toxicity was the primary reason for the dose reduction. No statistically significant difference was found in the incidence of any dose reduction between the HP group and the HX group. Treatment failure in both groups was mainly due to disease progression (n = 32, 66.7% in the HP group and n = 28, 63.6% in the HX group), followed by toxicity (n = 11, 22.9% in the HP group and n = 9, 20.5% in the HX group).

### Efficacy and Survival

The ORR was 58.3% in the HP group (95% confidence interval [CI]: 44.4%–72.3%), including 2 CRs and 26 PRs; the ORR was 59.1% in the HX group (95% CI: 44.6%–73.6%), including 2 CRs and 24 PRs; however, no significant difference was observed between the 2 groups (odds ratio = 0.97, 95%CI: 0.42–2.23, *P* = 1.00). The response rate (RR), including CR, PR, and stable disease, was 83.3% in the HP group (95% CI: 72.8%–93.9%) and 84.1% in the HX group (95% CI: 73.3%–94.9%); no statistically significant difference was found in the RR between the 2 groups (odds ratio = 0.95, 95% CI: 0.31–2.87, *P* = 1.00) (Table 2).

**TABLE 2:**
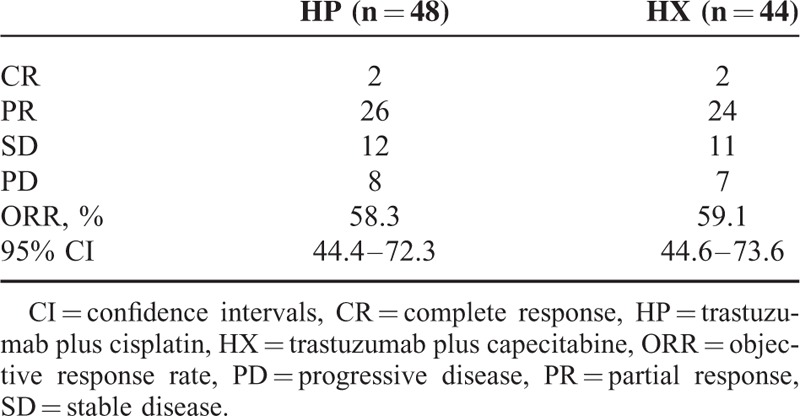
Response Rate in Each Group

**FIGURE 2:**
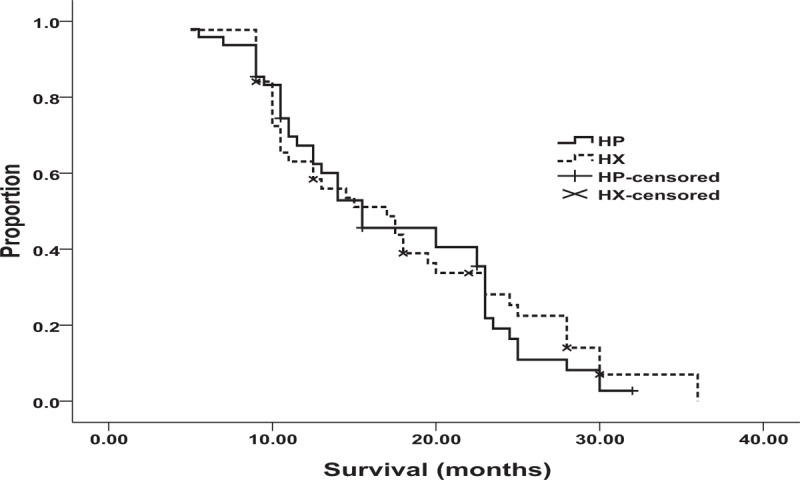
Overall survival in the HP group (n = 48): 15.5 months; in the HX group (n = 44): 17.0 months, (*P* = 0.78). HP = trastuzumab plus cisplatin, HX = trastuzumab plus capecitabine.

**FIGURE 3:**
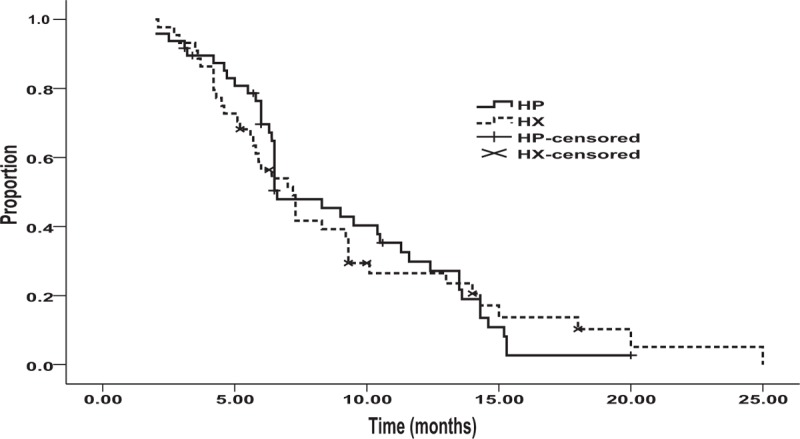
Progression-free survival in the HP group (n = 48): 6.6 months; in the HX group (n = 44): 7.2 months, (*P* = 0.90). HP = trastuzumab plus cisplatin, HX = trastuzumab plus capecitabine.

During the time period of the study, the median OS was 15.5 months in the HP group (95% CI: 10.2–20.4 months) and 17.0 months in the HX group (95% CI: 11.4–22.6 months) with no statistical significant difference between the groups, according to univariate analysis (hazard ratio 1.06, 95% CI: 0.68–1.66, *P* = 0.78) (Figure 2). The median PFS was 6.6 months (95% CI: 4.83–8.37 months) in the HP group and 7.2 months (95% CI: 5.88–8.52 months) in the HX group. The hazard ratio for disease progression or death (in both the HP and HX groups) was 0.97 (95% CI: 0.62–1.53, *P* = 0.90) (Figure 3). The estimated survival rate at 1 year was 56.3% in the HP group and 59.1% in the HX group; no statistically significant difference between the groups was found (relative risk = 1.07, 95% CI: 0.47–2.45). Similarly, there was no statistically significant difference in the estimated survival rates at 2 years in the HP group and in the HX group (12.5% vs 20.5%, respectively; relative risk = 1.1, 95% CI: 0.36–3.39).

**FIGURE 4:**
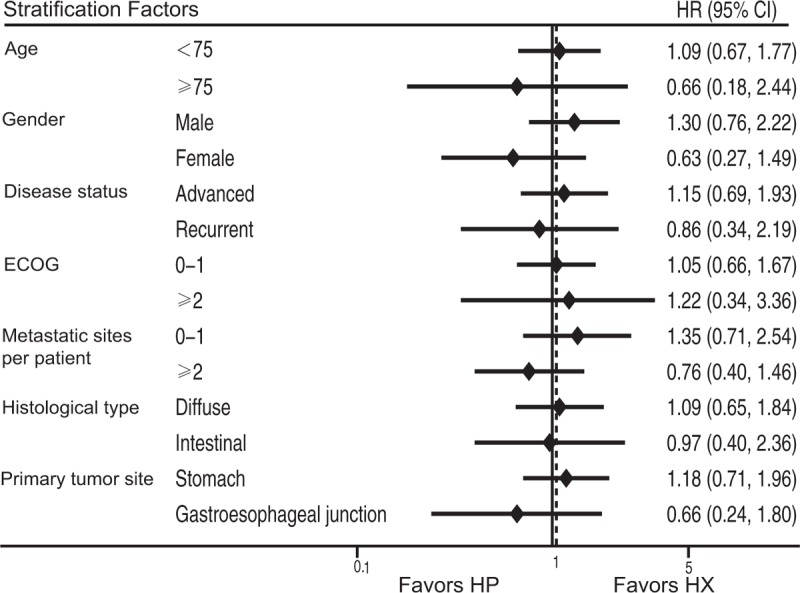
Subset analysis of OS for the HP and HX groups. HP = trastuzumab plus cisplatin, HX = trastuzumab plus capecitabine, OS = overall survival.

This study also conducted multivariate analyses of the predictive clinical factors for OS (Table 3). Age and histology were detected as independent prognostic factors using Cox regression analysis. In addition, subset analysis of OS indicated no apparent interaction between the clinical effects of each treatment (HP vs HX) and patient characteristics (Figure 4).

**TABLE 3:**
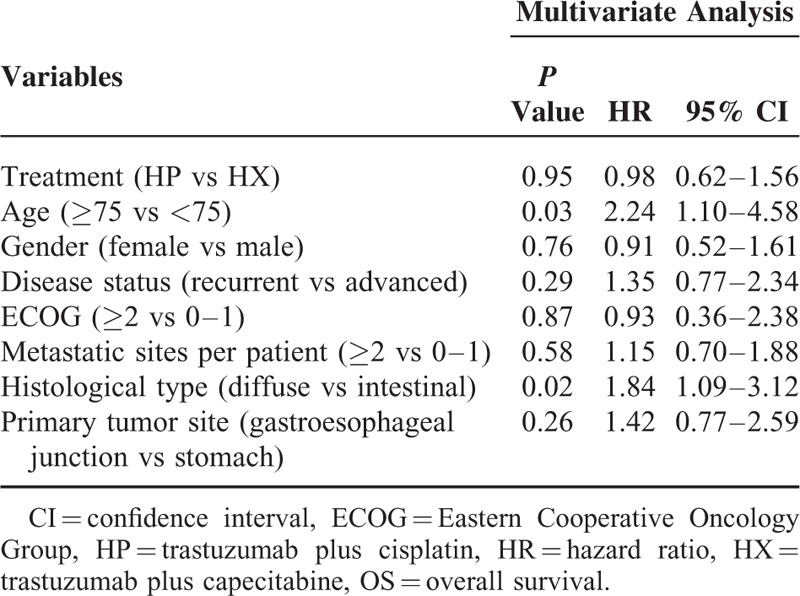
Multivariate Analysis of Predictive Clinical Factors for OS

### Toxicity

The adverse events observed in both groups are shown in Table 4. The most common hematological toxicity in both the HP group and HX group was leukopenia (68.8% vs 72.7%, respectively; *P* = 0.82). Nausea was the most frequent nonhematological toxicity in both the HP group and the HX group (68.8% vs 63.6%, respectively; *P* = 0.66). The most common grade 3 or 4 hematological toxicity and grade 3 or 4 nonhematological toxicity were neutropenia (HP: 35.4%, HX: 29.5%, *P* = 0.55) and anorexia (HP: 25.0%, HX: 22.7%, *P* = 0.80). Two patients (5.9%) in the HP group (due to grade 4 neutropenia) and 3 patients in the HX group (due to grade 3 anorexia) underwent a dose reduction. No treatment-related death occurred in either group.

**TABLE 4:**
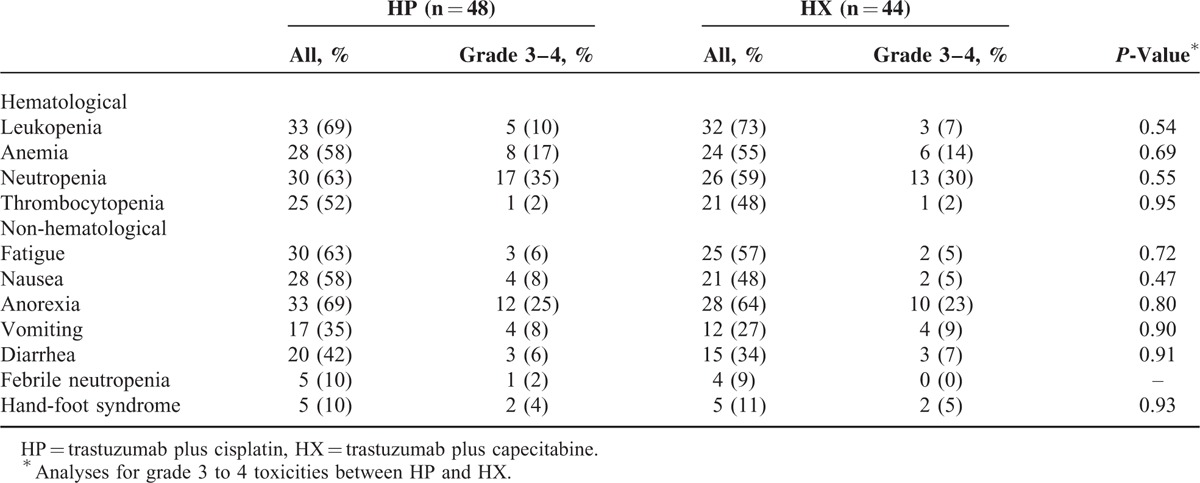
Toxicities

## DISCUSSION

This is the first retrospective trial that compared the treatment effect of first-line chemotherapy with HP or HX in elderly HER2-positive AGC patients. Our results indicated that HP and HX showed very similar efficacy in terms of OS, PFS, and ORR. No significant difference in toxicity, including any grade 3 to 4 adverse events, was found between the 2 groups. In addition, age and histology were detected as independent prognostic factors. Our conclusions suggest that either HP or HX can be considered as a first-line chemotherapy option for elderly HER2-positive AGC patients.

S-1 has already been confirmed to show promising anticancer efficacy for AGC. Results from a meta-analysis indicated that S-1-based therapy showed significant ORR, longer PFS, and longer OS than S-1 monotherapy, although with higher incidence of grade 3 to 4 neutropenia.^5^ In a retrospective trail that compared S-1 plus cisplatin (SP) to capecitabine plus cisplatin (XP), SP and XP were associated with similar efficacy and safety in AGC patients.^16^ However, for elderly HER2-positive ACG patients, trastuzumab might be a better treatment option than S-1.

Since trastuzumab has been demonstrated to show promising antitumor activity for HER2-positive breast cancer patients, trastuzumab alone, or in combination with other cytotoxic drugs, has been widely used in clinical practice to treat patients with that disease and most of the outcomes resulted in longer OS and PFS and higher ORR,^17–20^ even though that therapeutic approach is associated with a risk for cardiotoxicity.^21,22^ Trastuzumab-based adjuvant therapy has become the standard of care for HER2-positive breast cancer patients. The incidence of HER2-positive gastric cancer varies considerably between studies, ranging from 6.0% to 29.5%.^23–25^ The main reason for this diversification is that there has not been a recognized standard examination method and objective criteria for assessing HER2-status; moreover, in AGC patients, HER2 expression is also affected by the site of the primary tumor and histological type. Currently, HER2 status is mainly assessed by IHC and FISH protocols using biopsy or surgical specimen staining patterns.

The ToGA trial^26^ indicated that trastuzumab in combination with chemotherapy significantly improved OS in patients with high HER2 expression (ie, IHC 3+ or IHC 2+/FISH-positive) AGC or gastroesophageal cancer compared with chemotherapy alone. In addition, in that trial, the HER2-positive patients that received trastuzumab plus chemotherapy had longer OS than the HER2-negative patients who received the same regiment. It is also notable that trastuzumab did not increase the incidence of adverse events associated with chemotherapy and that the rate of cardiac events was low. A previous multicenter phase II study showed an ORR of 67%, 9.8 months median PFS, and 21.0 months median OS,^15^ which seems to be longer than the median OS of the patients in the HX group in our study. There could be several reasons for this. First, the median age of the patients included in that study^15^ was younger than the median age of the patients included in our trail (57 vs 71 years); in that study, the youngest patient was 29 and the oldest patient was 74 but in our trial the youngest patient was 66 and the oldest was 83. Subset analysis of the OS data from our trail indicated that age was considered to be an independent prognostic factor; this means that older patients experienced shorter OS. Second, patients in that study^15^ received oxaliplatin chemotherapy other than HX. Oxaliplatin is a cancer medication that interferes with the growth of cancer cells and works by killing cancer cells and slowing tumor growth. It has been considered to an effective anticancer drug to treat advanced colorectal cancer and other types of cancers. Therefore, a younger median age for the participants and the administration of an additional drug may have partially contributed to the improvements in the treatment effects. There was a treatment-related death case in that study,^15^ but none in our trail. In another phase II study,^14^ a combination of trastuzumab plus SP (S-1 plus cisplatin) showed a confirmed RR of 68%, a disease control rate of 94%, a 16.0-month median OS, a 7.8-month median PFS, and a 5.7-month intention-to-treat, which are similar to the treatment outcomes found with HP in our trail; therefore, a question was posed as to whether it is worth adding S-1 to the regimen used to treat HER2-positive AGC patients on the basis of the HP regimen findings. Although S-1 is a fluoropyrimidine preparation combining Tegafur with anticancer efficacy for AGC, it is still unknown whether HER2-positive AGC patient that received SP obtained longer survival than HER2-negative ACG patients that received the same regimen; however, it is known that an additional drug means a greater expenditure and more toxicity. As the participants in our current trail were elderly, a cisplatin dose of 60 mg/m^2^ instead of 80 mg/m^2^ was given because cisplatin is a well-known nephrotoxic drug.

In comparison to the patients in the HX group, more patients in the HP group had grade 3 to 4 adverse events (AEs), other than leukopenia and hand-foot syndrome. Although grade 3 to 4 AEs were more frequently observed in the HP group than in the HX group, no significant difference in AEs was found between the 2 groups.

Age and histology were detected as independent prognostic factors according to the results of a multivariate analysis of OS, which indicated that patients ≥75 years or with diffuse type of gastric cancer were considered to experience shorter OS that patients <75 years or with intestinal type of gastric cancer. From the results of the subset analysis of OS, no apparent interaction was found between the clinical effects of each treatment (HP vs HX) and patient characteristics, including age and histology.

Some limitations need to be taken into consideration when interpreting the findings. First, this study was a retrospective nonrandomized comparison; the process used to determine patient inclusion may raise the issue of selection bias, which may affect the results of our comparison. Second, patients who underwent second-line treatment, such as an S-1 agent, were excluded from the current study because second-line treatment is important for improving OS in HER2-positive AGC patients. Third, the small sample size from a single center is another limitation of this study.

In conclusion, although the retrospective nature of the study and the small number of patients are major limitations, the HP and HX treatment regimens were associated with similar efficacy and safety for HER2-positive AGC patients. Our conclusion needs to be confirmed via high-quality trials and the results need to be reproduced in other regions and populations.
